# Future Challenges in Psychotherapy Research for Personality Disorders

**DOI:** 10.1007/s11920-022-01379-4

**Published:** 2022-10-13

**Authors:** Ueli Kramer, Catherine F. Eubanks, Katja Bertsch, Sabine C. Herpertz, Shelley McMain, Lars Mehlum, Babette Renneberg, Johannes Zimmermann

**Affiliations:** 1 University of Lausanne, Lausanne, Switzerland; 2 University of Windsor, Windsor, Canada; 3 Adelphi University, Garden City, NY USA; 4 Ludwig-Maximilians-Universität Munich, Munich, Germany; 5 Universitätsklinikum Heidelberg, Heidelberg, Germany; 6 Centre for Addiction and Mental Health (CAMH) and University of Toronto, Toronto, Canada; 7 National Centre for Suicide Research and Prevention, Institute of Clinical Medicine, University of Oslo, Oslo, Norway; 8 Freie Universität Berlin, Berlin, Germany; 9 University of Kassel, Kassel, Germany

**Keywords:** Personality disorders, Psychotherapy research, Challenges, Methodology, Recommendation, Dimensional conception

## Abstract

**Purpose of Review:**

Individuals with personality disorders are frequently seen in mental health settings. Their symptoms typically reflect a high level of suffering and burden of disease, with potentially harmful societal consequences, including costs related to absenteeism at work, high use of health services, ineffective or harmful parenting, substance use, suicidal and non-suicidal self-harming behavior, and aggressiveness with legal consequences. Psychotherapy is currently the first-line treatment for patients with personality disorders, but the study of psychotherapy in the domain of personality disorders faces specific challenges.

**Recent Findings:**

Challenges include knowing what works for whom, identifying which putative mechanisms of change explain therapeutic effects, and including the social interaction context of patients with a personality disorder. By following a dimensional approach, psychotherapy research on personality disorders may serve as a model for the development and study of innovative psychotherapeutic interventions.

**Summary:**

We recommend developing the following: (a) an evidence base to make treatment decisions based on individual features; (b) a data-driven approach to predictors, moderators, and mechanisms of change in psychotherapy; (c) methods for studying the interaction between social context and psychotherapy.

## Introduction

Personality disorders (PDs) may be understood as pervasive, inflexible, and enduring patterns of experience or behavior that deviate markedly from common expectations, causing significant disturbances in self- and interpersonal functioning. Symptoms associated with PDs reflect a high level of suffering and burden of disease. As a consequence, high costs related to absenteeism at work, high use of health services, ineffective or harmful parenting, substance use, suicidal and non-suicidal self-harming behavior, and aggressiveness with legal consequences are reported. In the absence of effective PD-specific psychopharmacological treatments, psychotherapy remains the first-line treatment [1, 2••]. However, despite meta-analytic evidence demonstrating the probable efficacy (and effectiveness) of psychotherapy for PDs, key questions remain unanswered. To date, the majority of psychotherapy studies for patients presenting with PDs have either focused on (a) internally controlled comparative trials between two or more treatments (i.e., bonafide vs treatment as usual) and (b) pre- and post-comparisons of treatments as they are offered in the community. While valuable, such classical designs only partially address the pressing challenges the field of PDs currently faces. While the evidence base for psychotherapy is good for the average case, a significant portion of patients has a poor response to treatment [1, 2••]. A limitation of the current evidence base for psychotherapy for PDs is that it is largely restricted to patients with borderline personality disorder (BPD), but neglects the array of other personality pathology [1]. Also, it remains insufficiently clear what type of treatment works for what type of patients and this knowledge would enable clinicians to make differentiated treatment recommendations for individual patients [3]. There is a rudimentary knowledge base on the mechanisms of change in psychotherapy for patients with PDs, as well as an insufficient inclusion of social contextual factors for explaining recovery in PDs. It is necessary to address these challenges to further the development and dissemination of effective treatment methods and support the adequate treatment of large patient populations across the globe.

Consolidating evidence in this field will also need to rest on convincing study replications, which require stronger national and international collaborations between psychotherapy researchers. Researchers in Europe have formed a collaborative alliance to address the need for accelerating research on and dissemination of PD-specific treatments on this continent [4]. The scientific society under which the collaborative alliance is formed organizes training seminars for early career psychotherapy researchers interested in PDs to promote high-quality research in more European countries and offers workshop conferences for clinicians to accelerate the dissemination of evidence-based treatments in underserved regions. Finally, the alliance organizes research congresses and offers members and affiliates opportunities to develop their research collaboration on specific topics through an increasing number of thematic sections. Hopefully, such alliances will lead to a more systematic development of the research base needed to deliver affordable treatments in a sustainable and equitable way to the people who need them.

This paper, written by an expert group from this alliance, elaborates on the following three pressing challenges to psychotherapy research for PDs: (a) how can psychotherapy research determine what works for whom and thus make individualized treatment recommendations based on evidence? (b) How can psychotherapy research contribute to the knowledge of how and why change in PDs occurs? (c) How can psychotherapy research take into account the social (i.e., interpersonal) context of the treatment of PDs? A brief conclusion follows with recommendations for researchers on methodological approaches that may ultimately improve the impact of psychotherapeutic interventions for PDs.

## Developing an Evidence Base to Make Differentiated Recommendations on What Works for Whom

Typically, the efficacy of a particular treatment package is investigated using randomized controlled trials (RCTs), in which patients are randomly assigned to a treatment condition and a control condition. To date, treatment trials have largely been able to demonstrate the overall effectiveness of interventions in relatively heterogeneous clinical samples. However, only a few studies have collected samples large enough to allow for the analysis of subgroups or treatment moderators [5••]. Such analyses are needed to give us more precise information on who is benefiting the most from the treatments. Tailoring or optimizing treatments to individual patients’ needs is recommended in most treatment guidelines for people with PD [6], but to do so, we need more detailed and reliable knowledge. In order to determine what works for whom, we need evidence-based models that (a) describe psychopathology reliably and validly and (b) assess change due to psychotherapy by taking into account individual trajectories. Dimensional conceptions of PDs may help for both points. Consequences related to using dimensional conceptions concern (a) the selection of patients on the basis of inclusion and exclusion criteria, (b) the measurement of outcome variables, and (c) the statistical analysis of the results. Dimensional conceptions of PDs represent unique opportunities for addressing these current and future challenges.

When defining a treatment trial target population, a major problem with a categorical diagnostic approach is the issue of co-occurring disorders, i.e., that the individuals who meet the criteria for a particular disorder often also meet the criteria for other disorders. For example, approximately 74% of individuals diagnosed with BPD also meet the criteria for another PD [7], and about 85% of these individuals fulfill the criteria for another mental disorder [8]. As Hopwood et al. (2020, p. 3)[9] noted, in such a situation, “the researcher either has to select a patient population that is so specific as to be rare in actual practice (if the selection criterion is meeting a single diagnosis and no others) or so diverse that the interpretability of findings is imprecise (if multiple diagnoses are allowed).” In research on psychotherapy for BPD, the latter is probably true, as patients with co-occurring disorders are usually not excluded (with a few exceptions, such as comorbid psychotic disorder, bipolar disorder, and substance use [2••]). Considering the observation that the diagnosis of BPD itself encompasses heterogeneous symptoms, the implication is that the study results refer to a group of individuals with potentially very different problems. This makes it more difficult—although not impossible—to demonstrate the effectiveness of specific interventions or mechanisms of change at a group level. To address this problem, one approach is to accept the substantial heterogeneity of individual problems and base common treatment principles of the intervention on the severity of the problems and functional impairments [10–12]. The inclusion criterion would then not be a specific PD diagnosis but instead on a specific severity spectrum (e.g., severe PD according to ICD-11). An alternative possibility is to select patients based on the nature of their problematic traits [12–14]. Especially for mild or moderate PD’s according to ICD-11, it seems realistic to find patients who only fulfil the criteria for a certain trait domain but not for other trait domains. In more homogeneous groups of patients, it might be more feasible to demonstrate the efficacy of specific interventions or mechanisms of change.

A second problem with using a categorical understanding of mental disorders is that it frequently leads to the use of outcome measures that use arbitrary thresholds to classify individuals as recovered or remitted. For example, in research on BPD, a person who met four criteria over a period of time would be considered “remitted,” but a person who met five criteria would not [15]. This dichotomization loses important information about fine-grained differences in severity. In fact, Markon et al. [16] demonstrated that this reduces reliability by an average of 15% and validity by 37%, which in turn has unfavourable impact on the power of RCTs to detect treatment effects. To address this problem, the characterization of patients can include a map of the individual’s disorder-specific symptomatology and a broad multidimensional profile, because a dimensional understanding of disorders “makes no a priori assumptions about the level of the hierarchy at which a treatment might have its effects” ([9], p. 11). In this way, one could empirically determine which interventions have broad effects at the level of the general factor of PD and which are more likely to lead to specific changes in individual symptoms or facets.

Third, regarding statistical modeling, a categorical understanding of mental disorders typically leads to comparisons of the proportion of patients recovered or remitted between treatment conditions using statistical methods at the group level. This is problematic in two respects: on the one hand, it ignores the fact that outcome measurement is not perfect and is subject to measurement error; on the other hand, this way only the average effect of the treatment is estimated and the heterogeneity of individual treatment effects remains undetected. To address this problem, a dimensional definition of PD can facilitate the use of statistical models with latent variables to evaluate the effect of psychotherapy. Consequently, measurement error in individual indicators is explicitly accounted for and treatment effects can be estimated without measurement error. Provided that the relevant baseline covariates are included in the model and the sample is large enough, this approach allows for an estimate of the average treatment effect as well as an evaluation of the extent to which individual treatment effects depend on the specific characteristics of the person [17]. To some extent, specific studies in the recent meta-analysis by Storebø et al. [2••] tackled this problem for the diagnosis of BPD, although a broader approach is needed. A dimensional understanding of PD has the potential to achieve an evidence-based personalization of therapeutic interventions.

The notion of the functional domain (e.g., aggressiveness; [18••]) is a particularly fruitful avenue for re-conceptualizing PD and is related to dimensional models. Studies targeting functional domains advance psychotherapy research in several ways. A functional domain may be conceptualized as (a) an outcome criterion that complements symptom relief or (b) a variable that predicts change or moderates treatment effects, and (c) a mechanism of change in treatments. Importantly, change in functional domains may be observable on psychological and neurobiological levels [19, 20]. More research is needed to learn whether the dimensional constructs defining PDs may parallel specific functional domains ([21], e.g., reward responsiveness and valuation, and their relationship with the disinhibition trait domain [22]). Such re-conceptualization of PDs may have an impact on treatment content [14], length [23], and format of delivery [24].

## Developing an Evidence Base that Explains Why and How Psychotherapy Leads to Change

A pressing issue in psychotherapy research is the explanation of why and how psychotherapy leads to change in outcomes and, thus, the development of an evidence base that supports conceptualizations of mechanisms of change in psychotherapy. The study of mechanisms of change helps to advance an integrated model of change, which focuses less on theory-specific mechanisms and more on providing therapists with the knowledge they need to foster common mechanisms of change in a person-specific way [25].

A central mechanism of change in therapies for PDs may be building, maintaining, and repairing the therapeutic alliance. The therapeutic alliance, commonly defined as the patient and therapist’s agreement on therapy goals, collaboration on therapy tasks, and affective bond of trust and respect [26], is widely recognized as a crucial component of psychotherapy that bears a robust association with outcome across patient populations, including patients with PD diagnoses [27]. Many have noted the challenges of building and maintaining strong alliances with patients whose disorders are in part defined as disorders of interpersonal functioning (e.g., [28]). Hence, research on difficulties in the alliance, also known as alliance ruptures, is particularly relevant for therapy with patients with personality pathology. Studies have found that therapy with patients with PD diagnoses is marked by more alliance ruptures [29, 30] and ruptures of greater intensity [31] than comparison conditions of patients who do not have PD diagnoses.

Ruptures present challenges for treatment, as they mark moments of poor patient-therapist collaboration and, if left unrepaired, predict premature dropout and poor treatment outcome; at the same time, rupture repair is associated with good treatment outcome, and this association is not moderated by patient PD diagnosis [32]. Repairing an alliance rupture can provide a valuable corrective experience for patients who may be accustomed to experiencing criticism, rejection, or abandonment in their interpersonal relationships [33].

Other theorized mechanisms of change in treatments of PDs include improvement in emotion regulation (i.e., emotion awareness and transformation), coping skills, and changes in reflective capacities (i.e., mentalization, theory of mind). Research provides preliminary support for these three additional classes of mechanisms of change [34••, 35]. Several studies show that improvements in various facets of emotion regulation, including access to, awareness of, and experience of emotions, may be active ingredients of effective psychotherapy for patients with PDs (for review and discussions, [36••]) [11, 37]. Evidence suggests that the enhancement of specific mental activities, mentalizing, meta-cognitive and reflective capacities and schema modes may explain the psychotherapeutic change for PDs [34••]. Accumulating evidence also demonstrates that the enhancement of coping skills and improvements in mindfulness, especially a nonjudgmental stance, may constitute mechanisms of change [38•].

Most studies on mechanisms of psychotherapeutic change have attempted to validate specific theory-driven mechanisms; few studies have addressed the question of whether theorized treatment-specific mechanisms may be common across diverse approaches. A recent study by Euler et al. [39] explored whether a theorized non-treatment-specific mechanism of change in dialectical-behavior therapy (DBT)—change in defense mechanisms—explained the effects of DBT skills training for BPD. Improvements in overall defensive functioning were greater for those randomized to 20 weeks of DBT skills compared to those randomized to treatment as usual. The results indicate that a theorized treatment-specific mechanism (i.e., defensive functioning that is central to psychodynamic psychotherapy) may be a transtheoretical mechanism of psychotherapeutic action.

Most research to date has been based on simple correlational designs. There is a need for research that examines more complex temporal relationships between variables, as this may point to more specific treatment targets. A study by Kramer et al. [40] addressed this question by exploring the temporal relationship between clients’ behavioral coping and outcome. Based on a sample of 57 participants with BPD enrolled in 10 sessions of either good psychiatric management (GPM) or GPM combined with motive-oriented relationship (MOTR), the results showed that decreases in in-session behavioral coping between sessions 1 and 5 mediated outcomes measured between sessions 5 and 10, suggesting that preceding changes in coping may explain subsequent symptom change in brief therapy.

In designing studies on mechanisms of psychotherapeutic change, researchers should consider the inclusion of multiple process measures as well as adopt carefully planned temporal designs to allow for the exploration of the temporal relationship between variables [41••, 42••]. No studies to date involve the experimental manipulation of mechanisms, which limits causal conclusions [43, 44]. As the field moves forward, such studies are needed.

A final challenge when developing an evidence base on how and why psychotherapy produces its effects concerns the study of neurobiological correlates of mechanisms of change. Rather than broad treatment programs, future psychotherapies for patients with PDs may consist of treatment modules that target specific active ingredients in change [45]. To identify these active ingredients, psychologically informed neuroscience may provide a coherent framework to better understand the neuronal correlates that underlie changes in distinct psychological processes, such as cognition and emotion, motives, competencies, or habits. These different facets of the mind are rooted in various basic brain systems that may need to be specifically targeted and changed. Neuroscientific knowledge could thus be important for selecting targeted interventions since specific techniques affect different brain systems [46].

Several neuroimaging studies have investigated neuronal correlates of mechanisms of psychotherapy-induced change in emotion (or affective) dysregulation, one of the core functional impairments in BPD [47]. For instance, a study by Schmitt et al. [48] focused on the effects of DBT on improving reappraisal, the most efficient affect regulation strategy in daily life. The experiment asked participants to reduce their negative affective responses by reappraising the meaning of aversive social cues. Comparing patients who showed a treatment-response to DBT to nonresponders as well as a BPD control group who did not receive DBT, DBT responders exhibited reduced neural activities in the right amygdala, the rostral and dorsal anterior cingulate cortex, and in the orbitofrontal and dorsolateral prefrontal cortex after treatment. In addition, responders showed increased connectivity within the limbic-prefrontal network. Notably, the pre/post reduction of insula activity among DBT patients correlated with a reduction in affect dysregulation, suggesting a relationship between clinical improvement and lower insular activity as a core region of the salience system.

Threat hypersensitivity is a major explanatory construct of reactive aggression in BPD. In a recent randomized controlled trial [49••], researchers investigated whether a mechanism-based anti-aggression group psychotherapy (MAAP) with interventions that specifically target threat hypersensitivity was superior to a nonspecific supportive group psychotherapy (NSSP). Results indicated a better clinical outcome and reduced amygdala activity in response to facial emotions after MAAP, whereas amygdala activity was increased after NSSP. Furthermore, MAAP, but not NSSP, was followed by increased functional connectivity between the amygdala and dorsomedial prefrontal cortex from pre- to post-treatment [20].

Neuroscientific methods have helped to increase our understanding of how psychotherapy works. However, so far, experimental paradigms are rather removed from real life or real psychotherapy. More ecologically valid paradigms are therefore needed, which also vary the context in which symptoms are likely to occur. In the long run, neuroscience needs to include methods that can be transferred to the natural context. Furthermore, multimodal imaging has to date provided information on group effects rather than single subjects, and a more patient-centered approach may facilitate efforts to personalize treatment.

## Developing an Evidence Base on How Change Due to Psychotherapy is Embedded in a Social Interaction Context

In this final section, we consider how behavior change is influenced by the social interaction context. Family and friends of individuals with PD are often burdened by the interpersonal problems inherent to personality pathology [50]. Research on social functioning will enhance knowledge of these interpersonal dysfunctions and processes. However, a major challenge for research on social factors and interpersonal dysfunction is the sheer number of aspects that have been shown to be associated with the development and course of mental disorders as well as their complex interplay [51]. General factors include socioeconomic status, working and living conditions, informal and formal social supports, social network, social integration, as well as the quality of interpersonal relationships in vocational and personal life.

Focusing on social transactions in relationships, expectations, and first impressions of interactions with strangers were examined in participants with BPD [52]. Results show that compared to control groups, patients with BPD form more negative first impressions of others and are at the same time perceived more negatively by others. These processes might lead to reduced approach behavior and less positive reciprocal interactions on both sides, thereby contributing to conflictual relationships in patients with BPD [52]. Miano et al. [53] examined interactional behavior in romantic relationships and pointed to the intertwined problems of emotion regulation and interpersonal conflicts. A specific challenge in successfully treating mental disorders is the social context, including interpersonal patterns that are transmitted over generations. In order to break the cycle of transmission, it has to be considered if patients with children need help with parenting [54, 55]. An ongoing study is evaluating parenting training for mothers with BPD and their children [56, 57•]. An example of studying such social factors is the examination of the quality of youth welfare services provided to families of parents with a diagnosis of BPD.

Research on how social context impacts response to the treatment of PD poses specific methodological challenges. First, the assessment of social context variables should typically not be restricted to self-report methods, including observer raters of the individual’s behaviors. There is a need for longitudinal studies and the necessity of complex designs helping to disentangle the transactional nature of the social context. Examples of such complex designs are round robin or half-block designs, in which groups of participants make repeated assessments as they interact [58]. Based on the complex dyadic data thus obtained, researchers can analyze the perceptions, affective experiences, and interpersonal behaviors of individuals with PDs, as well as the reactions these individuals evoke in others [59]. In this regard, intensive longitudinal designs that take into account the social context of the individual (e.g., electronically activated recorder; EAR; [60]), as well as just-in-time adaptive interventions (JITAI [61]), may prove valuable.

In order to improve the course of recovery from PDs, more research on the social context and social transaction is needed. Research may inform psychotherapists’ efforts to improve tailored interventions [52, 53]. Additionally, we also need more studies investigating the impact of living conditions and patient-related outcomes in everyday life. Outcomes listed in the International Classification of Functioning, Disability, and Health (ICF) should be included in research studies. The importance of educating significant others about helpful ways of interacting has been pointed out, but systematic studies addressing the benefits are lacking. A related aspect is to increase public participation by including patients and relatives in the research. Examples include providing psychoeducation for families and carers of patients with PDs [62, 63]. Co-designing research that is both rigorous and inclusive is critical [48]. The development of interventions to improve social integration and social functioning will help patients and may at the same time serve as a preventive measure for their offspring. Relatedly, recent advances have highlighted the necessity of embedding psychotherapeutic interventions into specific cultural contexts, including minority status and gender [64].

## Conclusions and Research Recommendations

Evidence-based treatments for PD are effective under certain circumstances, which have started to be elucidated, and more research is needed. In order to address the pressing issues in the domain of psychotherapy for PDs, we discussed the need to refer to a dimensional classification which may increase the precision of outcome studies and help to develop knowledge on what works for whom. In order to advance the understanding of psychotherapy for PDs, we discussed the study of mechanisms of change from an integrative viewpoint and proposed novel designs addressing the limitations of earlier studies, including the essential assessment of biological correlates. In order to take a broad picture of recovery in the context of psychotherapy for PDs, we argued that researchers should include social context variables, including variables pertaining to a social transaction, in an informed and balanced way.

This paper highlights challenges in the field of psychotherapy research for PDs and recommends solutions. Our proposed solutions recommend moving beyond classical comparator designs toward research that accounts for the complexity of PDs, the processes of change in psychotherapy, and the social context within which psychotherapeutic change takes place. This requires moving away from group-based comparisons toward more fine-grained comparisons of change, which may become increasingly relevant to the individual.

In conclusion, we put forward three main recommendations for psychotherapy researchers. First, in order to assist with making treatment decisions, we recommend that researchers develop an evidence base that is informed by individual responses. Second, in order to explain the effects of treatments, we recommend that researchers develop a data-driven approach to predictors, moderators, and mechanisms of change. Third, in order to get a broad picture of change in psychotherapy, we recommend that researchers study recovery in PDs by taking into account the social context, including social transactions.

In the future, in order to build such valid and robust knowledge related to either of these recommendations, researchers need to rigorously build on earlier existing studies and systematically broaden the evidence base. Solid and broadly valid knowledge is not necessarily built within a separate therapy approach (i.e., dialectical-behavior therapy or mentalization-based therapy) but should increasingly adopt a theory-integrative focus that aims at using concepts pertaining to a multitude of theories, clinical practices, and contexts. Preregistration, registered reports, and replication studies are becoming the norm in basic science [65] but remain rare in research on PDs [66], yet they are extremely important when it comes to building such solid and robust knowledge for the future. Outcome research may also increasingly use more advanced designs, such as the Sequential Multiple Assignment Randomized Trial (“SMART”; [67]). More research should aim at refining and consolidating conclusions from earlier studies.

In the future, in order to take into account the critical idiosyncratic characterizations of patients with PDs, it is useful to consider methods based on intensive longitudinal designs [68, 69], idiographic network analysis [70], and the development of case formulation methodologies [42••]. Such approaches should be implemented more rigorously in the future [71–73]. Only relying on nomothetic variables may overlook the more fine-grained idiographic dynamics and meaning structures that are increasingly accessible to quantitative assessment tools. Linking these manifestations to dimensional constructs of PDs [74], as well as to their neurobiological underpinnings, remain challenges ahead.

In the future, psychotherapy research for PDs may need to address clinically pressing questions even more. Among these is the necessity for large dissemination of evidence-based treatments for PDs, in order to make sure that those who need the treatment receive it. Psychotherapies for PDs tend to be lengthy (1 to 3 years) and intensive (several sessions per week) and rely heavily on access to highly skilled therapists. Even wealthy countries lack a supply of therapists skilled in evidence-based PD-specific treatments that can adequately meet public health demands [75]. Several attempts have been made to solve these challenges [23, 76], for example, by developing brief or simplified therapeutic approaches for PDs, as recently reviewed by Dixon-Gordon et al. [77]. Along these lines, several studies have examined whether DBT skills training of varying duration could be delivered as a stand-alone intervention while still retaining sufficient improvement in important treatment targets, such as reductions in suicidal and self-injurious behaviors. It appears that for an online version of such skills training—with unclear adherence to DBT principles—researchers reported an increase in the risk of self-harming behavior in at-risk adults [78], while in sufficiently adherent DBT protocols for patients with PDs and other pathologies, the DBT skills components have repeatedly been associated with a reduction in suicidal and para-suicidal behaviors and other relevant outcomes [77].

In order to address our research recommendations, it is important, but insufficient, to learn about the efficacy of a structured and complex package of intervention; there are clinically critical situations for which therapists need evidence-based and appropriate responses and where many therapy manuals may fall short of providing guidance. An example may be efforts to train therapists to identify alliance ruptures and address them using strategies such as therapeutic meta-communication [79]. A study found that introducing alliance-focused training to therapists working with patients with PDs facilitated decreases in negative interpersonal behaviors such as therapists blaming or controlling and patients submitting, and increases in positive behaviors such as therapists affirming and both patients and therapists expressing their inner experience [80]. Again, more research targeting specific functional domains when they emerge as problematic in a specific therapy process may be studied using “if-then” algorithms (i.e., “if the client does this, then the therapist should respond like that”).

To conclude, in the future, even more so than in the past given, the multi-faceted challenges the field faces, collaboration between researchers will be key. We advocate for international, multi-disciplinary, theory-integrative, and broad collaborations which aim at rigorously refining and consolidating of knowledge into robust models. Scientific societies may play a key role in this task. In the domain of PDs, several scientific societies, such as the European Society for the Study of Personality Disorders (ESSPD), play an important role in the creation of research networks of collaboration across Europe [4]. We hope that such initiatives may contribute to address some of the challenges described in the current synthesis.

## References

[1] Budge SL, Moore JT, Del Re A, Wampold BE, Baardseth T, Nienhuis JB (2013). The effectiveness of evidence-based treatments for personality disorders when comparing treatment-as-usual and bona fide treatments. Clin Psychol Rev.

[2] •• Storebø OJ, Stoffers-Winterling JM, Völlm BA, Kongerslev MT, Mattivi JT, Jørgensen MS, Faltinsen E, Todorovac A, Sales CP, Callesen HE, Lieb K, Simonsen E. Psychological therapies for people with borderline personality disorder. Cochrane Database Syst Rev. 2020;5:CD012955. 10.1002/14651858.CD012955.pub2. **Current Cochrane meta-analysis on the effectiveness of psychotherapy for patients with borderline personality disorder**. 10.1002/14651858.CD012955.pub2 PMC7199382 32368793

[3] Hagerty SL. Toward precision characterization and treatment of psychopathology: a path forward and integrative framework of the Hierarchical Taxonomy of Psychopathology and the Research Domain Criteria. Persp Psychol Sci. 2022;1–19. 10.1177/17456916221079597. 10.1177/17456916221079597 35867337

[4] Mehlum L, Bateman A, Dalewijk HJ, Doering S, Kaera A, Moran PA, Bohus M. Building a strong European alliance for personality disorder research and intervention. Borderline Personal Disord Emot Dysregul. 2018;5:7. 10.1186/s40479-018-0082-z. 10.1186/s40479-018-0082-z PMC5879813 29619226

[5] •• Keefe JR, Kim TT, DeRubeis RJ, Streiner DL, Links PS, McMain SF. Treatment selection in borderline personality disorder between dialectical-behavior therapy and psychodynamic psychiatric management. Psychol Med. 2020;51:1829–1837. 10.1017/S0033291720000550. **An example of a methodologically strong study on predictors of effects in psychotherapy for borderline personality disorders, from a comparative perspective**. 10.1017/S0033291720000550 32204742

[6] Simonsen S, Bateman A, Bohus M, Dalewijk HJ, Doering S, Kaera A, Mehlum L. European guidelines for personality disorders: Past, present and future. Borderline Personality Disord Emot Dysregul, 2019;6:9. 10.1186/s40479-019-0106-3. 10.1186/s40479-019-0106-3 PMC6530178 31143448

[7] Grant BF, Chou SP, Goldstein RB, Huang B, Stinson FS, Saha TD, Smith SM, Dawson DA, Pulay AJ, Pickering RP, Ruan WJ (2008). Prevalence, correlates, disability, and comorbidity of DSM-IV borderline personality disorder: Results from the wave 2 national epidemiologic survey on alcohol and related conditions. J Clin Psychiatr.

[8] Lenzenweger MF, Lane MC, Loranger AW, Kessler RC (2007). DSM-IV personality disorders in the National Comorbidity Survey Replication. Biol Psychiatr.

[9] Hopwood CJ, Bagby RM, Gralnick T, Ro E, Ruggero C, Mullins-Sweatt S, Kotov R, Bach B, Cicero DC, Krueger RF, Patrick CJ, Chmielewski M, DeYoung CG, Docherty AR, Eaton NR, Forbush KT, Ivanova MY, Latzman RD, Pincus AL, Zimmermann J. Integrating psychotherapy with the hierarchical taxonomy of psychopathology (HiTOP). J Psychother Integr. 2020;30(4):477–497. 10.1037/int0000156.

[10] Bach B, Simonsen S (2001). How does level of personality functioning inform clinical management and treatment? Implications for ICD-11 classification of personality disorder severity. Curr Opin Psychiatr.

[11] Kramer U, Timulak L. The emotional underpinnings of personality pathology: Implications for psychotherapy. Clin Psychol: Sci Pract, 2022. 10.1037/cps000080.

[12] Mullins-Sweatt SN, Hopwood CJ, Chmielewski M, Meyer NA, Min J, Helle AC Walgren MD. Treatment of personality pathology through the lens of the hierarchical taxonomy of psychopathology: Developing a research agenda. Pers Ment Health. 2019. 10.1002/pmh.1464. 10.1002/pmh.1464 PMC7053295 31364820

[13] Hopwood CJ (2018). A framework for treating DSM-5 alternative model for personality disorder features. Personal Ment Health.

[14] Sauer-Zavala S, Southward MW, Hood CO, Elhusseini S, Fruhbauerova M, Stumpp N, Semcho S. Conceptual development and case data for a modular, personality-based treatment for borderline personality disorder. 2021. 10.31234/osf.io/mu3ez. 10.1037/per0000520 PMC9748867 35084872

[15] Zanarini MC, Frankenburg FR, Reich DB, Fitzmaurice G (2012). Attainment and stability of sustained symptomatic remission and recovery among patients with borderline personality disorder and axis II comparison subjects: a 16-year prospective follow-up study. Am J Psychiatr.

[16] Markon KE, Chmielewski M, Miller CJ (2011). The reliability and validity of discrete and continuous measures of psychopathology: a quantitative review. Psycholl Bull.

[17] Mayer A, Zimmermann J, Hoyer J, Salzer S. Wiltink J, Leibing E, Leichsenring F. Interindividual differences in treatment effects based on structural equation models with latent variables: an EffectLiteR tutorial. Struct Equat Model. 2020;27(5):798–816. 10.1080/10705511.2019.1671196.

[18] •• Bertsch K, Florange J, Herpertz SC. Understanding brain mechanisms of reactive aggression. Curr Psychiatr Rep. 2020;22(81). 10.1007/s11920-020-01208-6. **Review of human aggression as functional domain from a neurobiological perspective**. 10.1007/s11920-020-01208-6 PMC7661405 33180230

[19] Honecker H, Bertsch K, Spiess K, Krauch M, Kleindienst N. Herpertz SC, Neukel C. Impact of a mechanism-based anti-aggression psychotherapy on behavioral mechanisms of aggression in patients with borderline personality disorder. Front Psychiatr. 2021;5(12):689267. 10.3389/fpsyt.2021.689267. 10.3389/fpsyt.2021.689267 PMC8374952 34421676

[20] Neukel C, Bertsch K, Wenigmann M, Spiess K, Krauch M, Steinmann S, Herpertz SC. A mechanism-based approach to anti-aggression psychotherapy in borderline personality disorder: Group treatment affects amygdala activation and connectivity. Brain Sci. 2021;11(12):1627. 10.3390/brainsci11121627. 10.3390/brainsci11121627 PMC8699721 34942929

[21] Flechsenhar A, Kanske P, Krach S, Korn C. Bertsch K. The (Un)learning of social functions and its significance for mental health. PsyArXiv. 2022. Preprint: https://psyarxiv.com/bze7g. 10.1016/j.cpr.2022.102204 36216722

[22] Michelini G, Palumbo IM, DeYoung CG, Latzman, Kotov R. Linking RDoC and HiTOP: a new interface for advancing psychiatric nosology and neuroscience. Clin Psychol Rev. 2021;86. 10.1016/j.cpr.2021.102025. 10.1016/j.cpr.2021.102025 PMC8165014 33798996

[23] Kramer U, Kolly S, Charbon P, Ilagan GS, Choi-Kain LW. Brief psychiatric treatment for borderline personality disorder as a first step of care: Adapting general psychiatric management to a 10-session intervention. Pers Dis: Theor Res Treat. 2021. Advance online publication. 10.1037/per0000511. 10.1037/per0000511 34516155

[24] Kerber A, Schaeuffele C, Krieger T, Urech A, Riper H, Berger T, Boettcher J, Knaevelsrud C. Differential effects of psychological interventions in online and face-to-face settings on DSM-5 and ICD-11 maladaptive trait domains: an exploratory pilot study. Front Psychiatr. 2021;12:648367. 10.3389/fpsyt.2021.648367. 10.3389/fpsyt.2021.648367 PMC8236509 34194347

[25] Kazdin AE. Understanding how and why psychotherapy leads to change. Psychother Res. 2009;19(4–5):418–428. 10.1080/10503300802448899 19034715

[26] Bordin E. The generalizability of the psychoanalytic concept of the working alliance. Psychother: Ther Res Pract. 1979;16:252–260. 10.1037/h0085885.

[27] Flückiger C, Del Re AC, Wampold BE, Horvath AO (2018). The alliance in adult psychotherapy: a meta-analytic synthesis. Psychother.

[28] Benjamin LS, Karpiak CP. Personality disorders. Psychother: Theo Res Pract Train. 2001;38(4):487–491. 10.1037/0033-3204.38.4.487.

[29] Coutinho J, Ribeiro E, Fernandes C, Sousa I, Safran JD (2014). The development of the therapeutic alliance and the emergence of alliance ruptures. Anal Psicolog.

[30] Colli A, Gentile D, Condino V, Lingiardi V (2017). Assessing alliance ruptures and resolutions: Reliability and validity of the collaborative interactions scale-revised version. Psychother Res.

[31] Tufekcioglu S, Muran JC, Safran JD, Winston A (2013). Personality disorder and early therapeutic alliance in two time-limited therapies. Psychother Res.

[32] Eubanks CF, Muran JC, Safran JD (2018). Alliance rupture repair: a meta-analysis. Psychother.

[33] Christian C, Safran, JD, Muran JC. The corrective emotional experience: a relational perspective and critique. In LG Castonguay, CE Hill (Eds.), Transformation in psychotherapy: Corrective experiences across cognitive behavioral, humanistic, and psychodynamic approaches, 2012, pp. 51–67. American Psychological Association. 10.1037/13747-004.

[34] •• Kramer U, Beuchat H, Grandjean L, Pascual-Leone A. How personality disorders change in psychotherapy: a concise review of process. Curr Psychiatr Rep. 2020;22(41). 10.1007/s11920-020-01162-3. **A recent review on processes of change in psychotherapy for personality disorders**. 10.1007/s11920-020-01162-3 32519017

[35] Mehlum L, Ramleth R-K, Tormoen AJ, Haga E, Diep LM, Stanley BH, Miller AL, Larsson B, Sund AM, Groholt B (2019). Long term effectiveness of dialectical behavior therapy versus enhanced usual care for adolescents with self-harming and suicidal behavio*r*. J Child Psychol Psychiatr.

[36] •• Rudge S, Feigenbaum JD, Fonagy P. Mechanisms of change in dialectical behaviour therapy and cognitive behaviour therapy for borderline personality disorder: a critical review of the literature. J Ment Health 2020;29(1):92–102. 10.1080/09638237.2017.1322185. **A comprehensive review of mechanisms of change in dialectical-behavior therapy and cognitive-behavior therapy for patients with borderline personality disorders with a focus on emotion regulation**. 10.1080/09638237.2017.1322185 28480806

[37] Lincoln TM, Schulze L Renneberg B. The role of emotion regulation in the characterization, development and treatment of psychopathology. Nat Rev Psychol. 2022;1:272–286. 10.1038/s44159-022-00040-4.

[38] • Zeifman RJ, Boritz T, Barnhart R, Labrish C, McMain SF. The independent roles of mindfulness and distress tolerance in treatment outcomes in dialectical behavior therapy skills training. Perso Dis. 2019;11(3):181–190. 10.1037/per0000268. **An example of a mediation analysis studying mechanisms of change in psychotherapy for borderline personality disorder**. 10.1037/per0000368 31647267

[39] Euler S, Stalujanis E, Allenbach G, Koll S, de Roten Y, Despland JN, Kramer U (2019). Dialectical behavior therapy skills training affects defense mechanisms in borderline personality disorder – an integrative approach of mechanisms in psychotherapy. Psychother Res.

[40] Kramer U, Keller S, Caspar F, de Roten Y, Despland JN, Kolly S (2017). Early change in coping strategies in responsive treatments for borderline personality disorder. J Consult Clin Psychol.

[41] •• Falkenström F, Solomonov N, Rubel JA. How to model and interpret cross-lagged effects in psychotherapy mechanisms of change research: a comparison of multilevel and structural equation models. J Consult Clin Psychol. 2022;90(5):446–458. 10.1037/ccp0000727. **A statistical demonstration of cross-lagged effect as method for the study of mechanisms of change in psychotherapy (not in the domain of personality disorder)**. 10.1037/ccp0000727 PMC9245087 35604748

[42] •• Kramer U, Ranjbar S, Caspar F. Using case formulation for prediction of the therapeutic alliance in treatment for borderline personality disorder. Pers Disord. 2022. 10.1037/per0000555. **A statistical demonstration of the use of cross-lagged methodology in the context of the impact of case formulation on the alliance process in treatments for borderline personality disorder**. 10.1037/per0000555 35511572

[43] Ehring T, Limburg K, Kunze AE, Wittekind CE, Werner GG, Wolkenstein L, Guzey M, Cludius B. (When and how) does basic research in clinical psychology lead to more effective psychological treatment for mental disorders? Clin Psychol Rev. 2022;95. 10.1016/j.cpr.2022.102163. 10.1016/j.cpr.2022.102163 35660924

[44] Rohrer JM, Murayama K. These are not the effects you are looking for: Causality and the within-/between-person distinction in longitudinal data analysis. PsyArXiv. 2022. Preprint: https://psyarxiv.com/tg4vj.

[45] Dombrowski SU, O'Carroll RE, Williams B (2016). Form of delivery as a key 'active ingredient' in behaviour change interventions. Br J Health Psychol.

[46] Valk SL, Bernhardt BC, Trautwein FM, Bockler A, Kanske P, Guizard N, Collins DL, Singer T (2017). Structural plasticity of the social brain: Differential change after socio-affective and cognitive mental training. Scie Adv.

[47] Marceau EM, Meuldijk D, Townsend ML, Solowij N, Grenyer BFS (2018). Biomarker correlates of psychotherapy outcomes in borderline personality disorder: a systematic review. Neurosci Biobeh Rev.

[48] Schmitt R, Winter D, Niedtfeld I, Herpertz SC, Schmahl C (2016). Effects of psychotherapy on neuronal correlates of reappraisal in female patients with borderline personality disorder. Biol Psychiatr Cogn Neurosci Neuroimag.

[49] •• Herpertz SC, Matzke B, Hillmann K, Neukel C, Mancke F, Jaentsch B, Schwenger U, Honecker H, Bullenkamp R, Steinmann S, Krauch M, Bauer S, Borzikowsky C, Bertsch K, Dempfle A. A mechanism-based group-psychotherapy approach to aggressive behaviour in borderline personality disorder: findings from a cluster-randomized controlled trial. B J Psych Open. 2020;7(1):e17. 10.1192/bjo.2020.131. **The demonstration of efficacy when using a modular, mechanisms-based approach to treat aggression in borderline personality disorder**. 10.1192/bjo.2020.131 PMC7791567 33308363

[50] Bohus M, Stoffers-Winterling J, Sharp C, Krause-Utz A, Schmahl C, Lieb K (2021). Borderline personality disorder. Lancet.

[51] Gartland D, Riggs E, Giallo R, Glover K, Casey S, Muyeen S (2021). Using participatory methods to engage diverse families in research about resilience in middle childhood. J Health Care Poor Underserv.

[52] Hepp J, Kieslich PJ, Schmitz M, Schmahl C, Niedtfeld I (2021). Negativity on two sides: Individuals with borderline personality disorder form negative first impressions of others and are perceived negatively by them. Pers Disord.

[53] Miano A, Barnow S, Wagner S, Roepke S, Dziobek I (2021). Dyadic emotion regulation in women with borderline personality disorder. Cogn Ther Res.

[54] Florange JG, Herpertz SC (2019). Parenting in patients with borderline personality disorder, sequelae for the offspring and approaches to treatment and prevention. Curr Psychiatr Rep.

[55] Renneberg B, Rosenbach C (2016). There is not much help for mothers like me: Parenting Skills for mothers with borderline personality disorder - a newly developed group training program. Borderl Pers Dis Emot Dysregul.

[56] Rosenbach C, Buck-Horstkotte S, Renneberg B. Parenting skills for mothers with BPD - a group training. 2020. https://refubium.fu-berlin.de/handle/fub188/26429. 10.17169/refubium-26189.

[57] • Rosenbach C, Heinrichs N, Kumsta R, Schneider S, Renneberg B. Study protocol for a multi-center RCT testing a group-based parenting intervention tailored to mothers with borderline personality disorder against a waiting control group (PROCHILD*-SP1). Trials. 2022. 10.1186/s13063-022-06531-2. **The protocol of an ongoing study on the effectiveness of training parents of children with borderline personality disorder**. 10.1186/s13063-022-06531-2 PMC9308114 35870944

[58] Kenny DA (2019). Interpersonal perception: the foundation of social relationships (Second edition).

[59] Rau R, Zimmermann J, Back MD. Applying multi-modal social relations analyses in personality pathology research. Person Disord. 2022. https://psyarxiv.com/n4sxz/. 10.1037/per0000589 36848075

[60] Mehl MR (2017). The electronically activated recorder (EAR): a method for the naturalistic observation of daily social behavior. Curr Direct Psychol Sci.

[61] Coppersmith DDL, Dempsey W. Kleiman EM, Bentley KH, Murphy SA, Nock MK. Just-in-time adaptive interventions for suicide prevention: promise, challenges and future directions. PsyArXiv. 2021. Preprint: 10.31234/osf.io/eg9fx. 10.1080/00332747.2022.2092828 PMC9643598 35848800

[62] Grenyer BFS, Bailey RC, Lewis KL, Matthias M, Garretti T, Bickerton A (2019). A randomized controlled trial of group psychoeducation for carers of persons with borderline personality disorder. J Person Disord.

[63] Sutherland R, Baker J, Prince S (2020). Support, interventions and outcomes for families/carers of people with borderline personality disorder: a systematic review. Person Ment Health.

[64] Harned MS, Coyle TN, Garcia NM (2022). The inclusion of ethnoracial, sexual, and gender minority groups in randomized controlled trials of dialectical-behavior therapy: a systematic review of the literature. Clin Psychol: Sci Pract.

[65] Nosek BA, Ebersole CR, DeHaven AC, Mellor DT (2018). The preregistration revolution. PNAS.

[66] Verschuere B, Yasrebi-de Kom FM, van Zelm I, Lilienfeld SO (2021). A plea for preregistration in personality disorders research: the case of psychopathy. J Person Disord.

[67] Lei H, Nahum-Shani I, Lynch K, Oslin D, Murphy SA. A “SMART” design for building individualized treatment sequences. Ann Rev Clin Psychol. 2012;8:21–48. 10.1146/annurev-clinpsy-032511-143152. 10.1146/annurev-clinpsy-032511-143152 PMC3887122 22224838

[68] Harpoth TS, Hepp J, Trull TJ, Bateman AW, Kongerslev MT, Simonsen E. Positive affect is associated with decreased symptom severity in the daily lives of individuals with borderline personality disorder. J Person Disord. 2019;1–18. 10.1521/pedi_2019_33_453 31682195

[69] Zimmermann J, Woods WC, Ritter S, Happel M, Masuhr O, Jeger U, Spitzer C, Wright AGC (2019). Integrative structure and dynamics in personality assessment: first steps toward the development and validation of a personality dynamics diary. Psychol Assess.

[70] Howe E, Bosley HG, Fisher AJ. Idiographic network analysis of discrete mood states prior to treatment. Counsell Psychother Res. 2020;20:470–478. 10.1002/capr.12295.

[71] Kamphius JH, Noordhof A, Hopwood CJ (2020). When and how assessment matters: an update on the treatment utility of clinical assessment (TUCA). Psychol Assess.

[72] Kramer U (2020). Individualizing psychotherapy research designs. J Psychother Integr.

[73] Lewis CC, Boyd M, Puspitasari A, Navarro E, Howard J, Kassab H, Hoffman M, Scott K, Lyon A, Douglas S, Simon G. Implementing measurement-based care in behavioral health: a review. JAMA Psychiatry. 2020. 10.1001/jamapsychiatry.2018.3329 PMC6584602 30566197

[74] Dotterer HL, Beltz AM, Foster KT, Simms LJ, Wright ACG (2020). Personalized models of personality disorders: Using a temporal network method to understand symptomatology and daily functioning in a clinical sample. Psychol Med.

[75] Iliakis EA, Sonley AKI, Ilagan GS, Choi-Kain LW. Treatment of borderline personality disorder: is supply adequate to meet public health needs? Psychiatr Serv. 2019;70(9):772–781. 10.1176/appi.ps.201900073. 10.1176/appi.ps.201900073 31138059

[76] Hutsebaut J, Willemsen E, Bachrach N, Van R (2020). Improving access to and effectiveness of mental health care for personality disorders: the guideline-informed treatment for personality disorders (GIT-PD) initiative in the Netherlands. Border Person Disord Emot Regul.

[77] Dixon-Gordon KL, Conkey LC, Woods SE. Brief therapeutic approaches for personality disorders. In The Cambridge handbook of personality disorders. 2020, pp. 477–494. New York, NY, US: Cambridge University Press.

[78] Simon GE, Shortreed SM, Rossom RC, Bec A, Clarke GN, Whiteside U, Richards JE, Penfold RB, Boggs JM, Smith J. Effect of offering care management or online dialectical behavior skills training vs usual care on self-harm among adult outpatients with suicidal ideation: a randomized controlled trial. JAMA. 2022;27(7):630–638. 10.1001/jama.2022.0423. 10.1001/jama.2022.0423 PMC8848197 35166800

[79] Muran JC, Eubanks CF. Performance under pressure: negotiating emotion, difference, and rupture. 2020. American Psychological Association. 10.1037/0000182-000.

[80] Muran JC, Safran D, Eubanks CF, Gorman BS. The effect of alliance-focused training on a cognitive-behavioral therapy for personality disorders. J Consult Clin Psychol. 2018;86:384–397. 10.1037/ccp0000284. 10.1037/ccp0000284 PMC5901896 29648858

